# The complete chloroplast genome of parasitic flowering plant *Monotropa hypopitys*: extensive gene losses and size reduction

**DOI:** 10.1080/23802359.2016.1155090

**Published:** 2016-03-28

**Authors:** Eugene V. Gruzdev, Andrey V. Mardanov, Alexey V. Beletsky, Elena Z. Kochieva, Nikolai V. Ravin, Konstantin G. Skryabin

**Affiliations:** Institute of Bioengineering, Research Center of Biotechnology of the Russian Academy of Sciences, Moscow, Russia

**Keywords:** Chloroplast genome, gene loss, *Monotropa hypopitys*, mycoheterotrophy, parasitic plant

## Abstract

Plastid genomes of parasitic plants represent apt systems, in which the effects of relaxed selective pressure on photosynthetic function are studied. The complete chloroplast genome sequence of nonphotosynthetic mycoheterotrophic plant *Monotropa hypopitys* was determined. With only 19 protein-coding, four rRNA and 17 tRNA genes in 34 800 bp long genome, it is one of the most reduced plastid genomes characterized until now. *Monotropa* chloroplast genome lacks all genes encoding photosynthetic functions and RNA polymerase subunits but retains most of the ribosomal protein genes and housekeeping genes infA and matK. *Monotropa* represents the late stages of chloroplast genome decay following the transition to heterotrophy.

The chloroplast genomes (cpDNAs) of most flowering plants contain a conserved set of about 110 genes, encoding photosynthesis apparatus, transcription/translation system and other housekeeping functions (Palmer 1985). Structurally, cpDNA comprises two single copy regions that are separated by two identical large inverted repeats. Genomic studies of nonphotosynthetic plants, which parasitize either other flowering plants or mycorrhizal fungi (mycoheterotrophs), revealed cpDNAs rearranged structurally and reduced in size and gene content (dePamphilis and Palmer 1990; Wicke et al. 2011). Mycoheterotrophs are relatively rare among angiosperms and comprise approximately 400 species (Merckx and Freudenstein 2010). Several full chloroplast genomes of mycoheterotrophic plants have been published, including the five orchid species: *Corallorhiza striata*, *Epigogium roseum*, *Epigogium aphyllum*, *Neottia nidus-avis* and *Rhizanthella gardneri* (Delannoy et al. 2011; Logacheva et al. 2011; Barrett and Davis 2012; Schelkunov et al. 2015), the monocots *Petrosavia stellaris* (Logacheva et al. 2014) and *Sciaphila densiflora* (Lam et al. 2015) and the liverwort *Aneura mirabilis* (Wickett et al. 2008). These cpDNAs represented different stages of plastid genome degradation.

In this study, we determined complete sequence of the chloroplast genome of a non-photosynthetic mycoheterotrophic plant *Monotropa hypopitys* (pinesap; *Ericaceae*), native to temperate regions of the Northern Hemisphere (Bidartondo 2005). A single *Monotropa hypopitys* plant was collected in Kaluga region, Russia (54°41′00″ N, 36°02′38″ E). The specimen is stored under accession number MON-2KALR. Total genomic DNA was extracted from fresh leaves of a single individual and sequenced using the GS FLX platform (Roche, Switzerland), with 8-kb paired end sequencing method. *De novo* assembly was performed with GS De Novo Assembler, which yielded a single chloroplast DNA scaffold with 103-fold coverage. The complete cpDNA sequence was obtained upon the generation and sequencing of appropriate PCR fragments. cpDNA annotation was performed using DOGMA (Wyman et al. 2004) with further manual correction. The cpDNA sequence of *M. hypopitys* was submitted to GenBank under accession number KU640958.

The 34 800 bp long chloroplast genome of *M. hypopitys* is one of the smallest sequenced cpDNAs, it lacks quadripartite structure typical to most of the other plastomes of angiosperms. The *M. hypopitys* cpDNA is predicted to contain 40 genes, including 19 protein-coding genes (ribosomal proteins; translation initiation factor *infA*; splicing factor *matK*), 4 ribosomal RNA genes as well as 17 transfer RNAs. Genes encoding NADH dehydrogenase, photosynthesis-related proteins, the plastid-encoded RNA polymerase are missing, as well as *clpP*, *ycf1* and *ycf2*. The *accD* ORF is highly diverged and probably non-functional. According to the model describing the pattern of gene loss during plastome degradation (Barrett et al. 2014), genes encoding transfer RNA and ribosome components and housekeeping genes *matK*, *clpP*, *infA*, *accD*, *ycf1* and *ycf2* are the last ones to be lost. The size and gene content of *M. hypopitys* cpDNA indicate that it is close to the end of plastid genome degradation process.

The maximum likelihood phylogenetic tree was based on concatenated sequences of chloroplast proteins from *M*. *hypopitys* and other 11 species (Figure 1). As expected, *M. hypopitys* is phylogenetically related to the family *Ericaceae* of the order *Ericales*.

**Figure 1.**
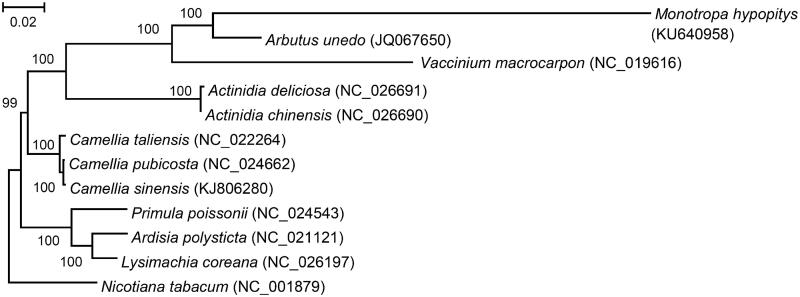
The maximum likelihood phylogenetic tree of *Monotropa hypopitys*, 10 other species from the *Ericales* order and *Nicotiana tabacum* as an outgroup. The tree is based on concatenated amino acid sequences of proteins, encoded by genes *rps12*, *rpl32*, *rpl23*, *rpl2*, *rps19*, *rpl22*, *rps3*, *rpl16*, *rpl14*, *rps8*, *infA*, *rpl36*, *rps11*, *rpl33*, *rps18*, *rpl20*, *rps4*, *rps14*, *clpP*, *accD*, *rps2*, *matK* and *rps7*. PhyML 3.1 (Guindon et al. 2010) was used for the sequence alignment and construction of the tree. Bootstrap support values are displayed on each node.
